# A Cosine Similarity-Based Method to Infer Variability of Chromatin Accessibility at the Single-Cell Level

**DOI:** 10.3389/fgene.2018.00319

**Published:** 2018-08-15

**Authors:** Stanley Cai, Georgios K. Georgakilas, John L. Johnson, Golnaz Vahedi

**Affiliations:** ^1^Department of Genetics, Perelman School of Medicine, University of Pennsylvania, Philadelphia, PA, United States; ^2^Penn Institute for Immunology, Perelman School of Medicine, University of Pennsylvania, Philadelphia, PA, United States; ^3^Penn Epigenetics Institute, Perelman School of Medicine, University of Pennsylvania, Philadelphia, PA, United States

**Keywords:** single-cell, chromatin, ATAC-seq, multidimensional scaling, variation, code:R

## Abstract

Cellular identity between generations of developing cells is propagated through the epigenome particularly via the accessible parts of the chromatin. It is now possible to measure chromatin accessibility at single-cell resolution using single-cell assay for transposase accessible chromatin (scATAC-seq), which can reveal the regulatory variation behind the phenotypic variation. However, single-cell chromatin accessibility data are sparse, binary, and high dimensional, leading to unique computational challenges. To overcome these difficulties, we developed PRISM, a computational workflow that quantifies cell-to-cell chromatin accessibility variation while controlling for technical biases. PRISM is a novel multidimensional scaling-based method using angular cosine distance metrics coupled with distance from the spatial centroid. PRISM takes differences in accessibility at each genomic region between single cells into account. Using data generated in our lab and publicly available, we showed that PRISM outperforms an existing algorithm, which relies on the aggregate of signal across a set of genomic regions. PRISM showed robustness to noise in cells with low coverage for measuring chromatin accessibility. Our approach revealed the previously undetected accessibility variation where accessible sites differ between cells but the total number of accessible sites is constant. We also showed that PRISM, but not an existing algorithm, can find suppressed heterogeneity of accessibility at CTCF binding sites. Our updated approach uncovers new biological results with profound implications on the cellular heterogeneity of chromatin architecture.

## Introduction

One of the great mysteries in developmental biology is how the same genome can be read by cellular machinery, giving rise to the plethora of different cell types required for eukaryotic life (Stunnenberg et al., 2016). The versatility of our genome is closely related to the myriad ways of packaging of DNA sequence into the chromatin, which is also referred to as the *epigenome*. Advances in sequencing technologies over the last decade enabled the genome-wide characterizations of the epigenome in many cell types and tissues. The availability of unbiased measurements of the chromatin suggest that the epigenome represents a second dimension of the genomic sequence and is pivotal for maintaining cell-type specific gene expression patterns.

Despite the inherently repressive state of the chromatin making most of the genome unreadable, in every cell type, a small segment is organized into cis-regulatory modules, known as promoters and enhancers, controlling the transcriptional output of the cell (Monticelli and Natoli, 2017). The emerging theme from recent studies is that the cis-regulatory landscape is organized hierarchically where every layer is regulated by distinct groups of transcription factors. The first layer consists of a small number of lineage-determining transcription factors, also referred to as pioneer factors, who act first on the chromatin and either alone or in cooperation with other proteins can access their binding sites even if they are inaccessible by nucleosomes through the recruitment of chromatin-remodeling enzymes and exposing the underlying DNA. Other layers in this organization are controlled by majority of transcription factors, which can bind to the primed and accessible chromatin regions (Garber et al., 2012; Vahedi et al., 2012; Ostuni et al., 2013), determining functional characteristics of every cell type.

Because the conventional population-average epigenomic measurements rely on thousands to millions of cells, it is unclear if transcription factors act similarly across individual cells. Although it is appreciated that gene expression is a fundamentally stochastic process (Raj and van Oudenaarden, 2008; Raj et al., 2010; Symmons and Raj, 2016), with randomness leading to cell-to-cell variations in mRNA and protein levels, much less is known about how randomness of transcription factor binding can be linked to cell-to-cell variations. Would the precise coordination of development demand transcription factors with certain biochemical properties to confer a deterministic impact on the chromatin of individual cells following a certain trajectory?

The advent of single-cell genomics, which has enabled unbiased profiling of the genetic and molecular states of ever-growing number of individual cells, paved the way to address these fundamental questions (Tanay and Regev, 2017). Whereas single-cell RNA sequencing (scRNA-seq) has been at the forefront of these methods, single-cell chromatin accessibility assays such as scATAC-seq are constantly being optimized for increasing throughput, robustness, and complexity (Buenrostro et al., 2015; Cusanovich et al., 2015, 2018; Corces et al., 2016; Lake et al., 2018; Preissl et al., 2018). Complexity is particularly challenging for scATAC-seq, which must target single-copy molecules and, unlike single-cell RNA-seq, cannot buffer partial sampling through the analysis of high-copy-number molecules (Tanay and Regev, 2017). Only 0, 1, or 2 reads can be generated from elements within a diploid genome. Hence, scarcity is an intrinsic feature of these types of measurements, which hinders studying the role of transcription factors in establishing the chromatin accessibility landscape at the single cell level. Thus, new computational methods incorporating the scarcity of data and the assay’s inherent bias are required.

The current leading method for measuring cell-to-cell variation from scATAC-seq measurements, chromVAR (Schep et al., 2017), measures total accessibility in an individual cell at a set of DNA sequences unified by a common feature. Total accessibility refers to the number of open chromatin regions in a cell. A common feature can be defined as binding events of a transcription factor, enrichment of a set of DNA binding motifs, or deposition of a combination of histone modifications. It then measures how much total accessibility differs from what is expected by calculating a technical bias-corrected *Z*-score for each cell. The standard deviation of these *Z*-scores constitutes the cell-to-cell variation in chromatin accessibility. However, an ensemble of cells can have similar total accessibility (i.e., number of accessible sites in a cell) yet be accessible at completely different regulatory elements. Thus, chromVAR is poorly equipped to handle chromatin accessibility variation in certain cases as stated in the original paper (Schep et al., 2017).

Here, we present PRISM, an R package for calculating cell-to-cell variation in chromatin accessibility using cosine similarity. Instead of measuring variation in total accessibility between two cells, PRISM measures whether two cells are accessible at the same set of regulatory elements using angular cosine distance. It then exploits principal coordinate analysis to measure how much each cell differs from the group norm for chromatin accessibility. Here, we demonstrate that PRISM outperforms chromVAR on various simulations when total accessibility is not varied, when signal is low, or when technical noise is high, in addition to real biological data. Together, PRISM can be used to construct a global and high-resolution view of epigenomic regulation in development and disease.

## Materials and Methods

### Inferring Chromatin Accessibility Variation Prior to Bias Correction

**FIGURE 1:**
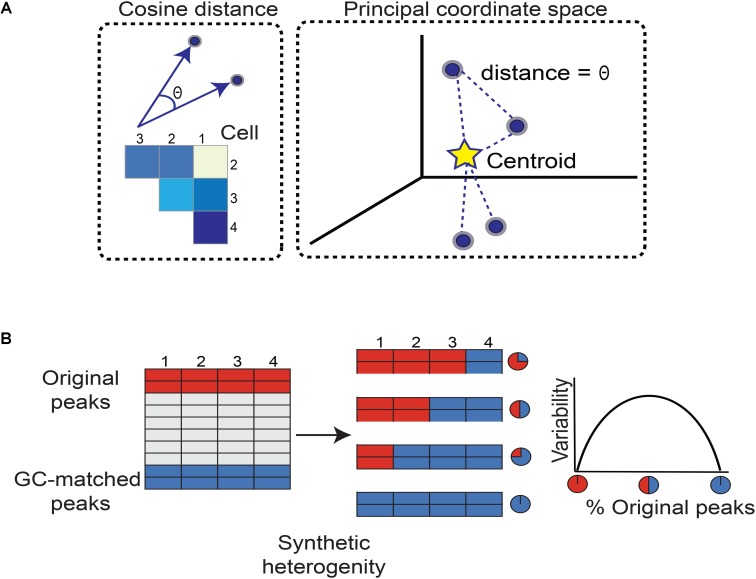
Workflow for quantifying chromatin accessibility variation and generating simulated heterogeneity. **(A)** To measure how different any two cells are in terms of chromatin accessibility, we measure the angle between their vectors. A larger angle implies the two cells are more different in chromatin accessibility. As there are many possible pairs of cells, we form a matrix of cosine distances between cells. To measure how variable the cells are as a whole with respect to accessible chromatin landscape, we perform principal coordinate analysis. Each cell is now plotted as a point in space. The Euclidean distance between any two cells is equal to the angle between their vectors. This specifies a unique point (cell) configuration in space and the centroid of the cells (points) is further calculated. Each cell’s distance from the centroid is measured. Then the average distance from each cell to the centroid constitutes the chromatin accessibility variation. Subsequent steps correct for technical biases. **(B)** We simulated heterogeneity within single cell chromatin accessibility maps using two models. While model 1 synthesizes heterogeneity assuming comparable levels of total accessibility between individual cells at a set of genomic regions, model 2 captures cell populations with large differences in total accessibility among cells. The simulated heterogeneity is then created by titrating the number of cells with the original or GC-matched peaks. This approach leads to a mixture of cells containing varying percentages of original versus GC-matched peaks. The rationale to mix peaks rather than cells of different types is to prevent confounding factors such as differences in cell lysis affecting our assessment of cell-to-cell variability. We expect variability to maximize when the data is a roughly 50–50 mixture of original and GC-matched peaks, and to minimize when data is completely original or GC-matched peaks, forming an inverse-U (concave down) shape.

PRISM requires the scATAC-seq data to be binarized such that accessible genomic regions are scored 1 and inaccessible ones are scored 0. Every cell is plotted as a vector with coordinates given by the binary representations of accessibility at a pre-defined set of genomic regions unified by a common characteristic – for example, binding events of a transcription factor measured by ChIP-seq or sequences enriched for transcription factor binding motifs (**Figure 1**). We then evaluate the difference in chromatin accessibility between a pair of cells at these genomic regions by calculating the cosine distance between every two vectors. More specifically, where *A* and *B* are binary accessibility vectors, the angular cosine distance is calculated by Equation (1), which can be seen as taking the angle between two vectors and dividing it by a normalizing factor of π/2:

(1) $$\text{Consine distance (}A,B\text{)=}\frac{{\text{cos}}^{-1}\frac{A\cdot B}{∥A∥\;\;\;∥B∥}}{\text{π}/2}$$

We calculate how different every cell is from the group norm and center the cosine distance matrix by subtracting column and row means while adding the overall mean. We then spectrally decompose the centered cosine distance matrix to define principal coordinates, mapping vectors of chromatin accessibility to full principal coordinate space, and identifying the vectors’ centroid (**Figure 1**). Finally, the average distance of all vectors to the centroid is used by PRISM as a measure of variability of chromatin accessibility among individual cells at the genomic regions of interest.

### Correcting for Technical Biases

The above formulation Equation (1) can be used to compare the impacts of different transcription factors in terms of inducing or reducing stochasticity of chromatin accessibility across individual cells. However, inherent differences among genomic subsets such as the GC content or average accessibility can also introduce technical variations. To overcome such limitations and normalize for the GC contents, we calculate variability as described in Equation (1) at a set of “normalizing background peaks” with GC contents comparable to the genomic set of interest. The normalizing background peaks are selected randomly from the set of genomic regions lacking the feature of interest based on their GC contents. The process of background peak selection is repeated for a user-defined number of times and the mean of variability for multiple sets of background peaks is used as a normalization factor. All background peaks are also selected to be within ±0.01 of the overall mean accessibility of the original peaks.

We controlled for technical biases as follows:

(2) $$\text{Bias corrected variation=}\frac{\text{Original variation}}{\text{Mean (background variations)}}$$

To measure accessibility variation beyond GC content, we calculate accessibility variation for a user-defined number of randomly selected subsamples of peaks lacking the genomic feature of interest, for example, regions not bound by a transcription factor while correcting for technical biases in the control peak set. Each subsample has the same number of peaks as the original peak set. The negative control variations are further used to generate *Z*-scores and *p*-values for the observed variation. The final variation is calculated by PRISM as:

(3) $$\text{Final variation=}\frac{\text{Bias corrected variation}}{\text{Mean (biased corrected negative control variations)}}$$

PRISM’s variability equal to 1 implies that the feature of interest for example a transcription factor or enrichment of a certain motif is associated with no more variation than negative control. PRISM’s variability below 1 implies that the feature of interest is associated with less variation than negative control, and variability above 1 implies greater variation than negative control. It is worth to point out that the idea behind PRISM is to assess the difference between cells at transcription factor-bound sites compared with the unbound regions (or regions lacking the genomic feature of interest). However, the two sets are not required to have identical GC-contents. To address this issue, the transcription factor-binding peak set and negative control peak set are each normalized for their GC-contents, which will require separate background peak selections.

### Benchmarking PRISM

**FIGURE 2:**
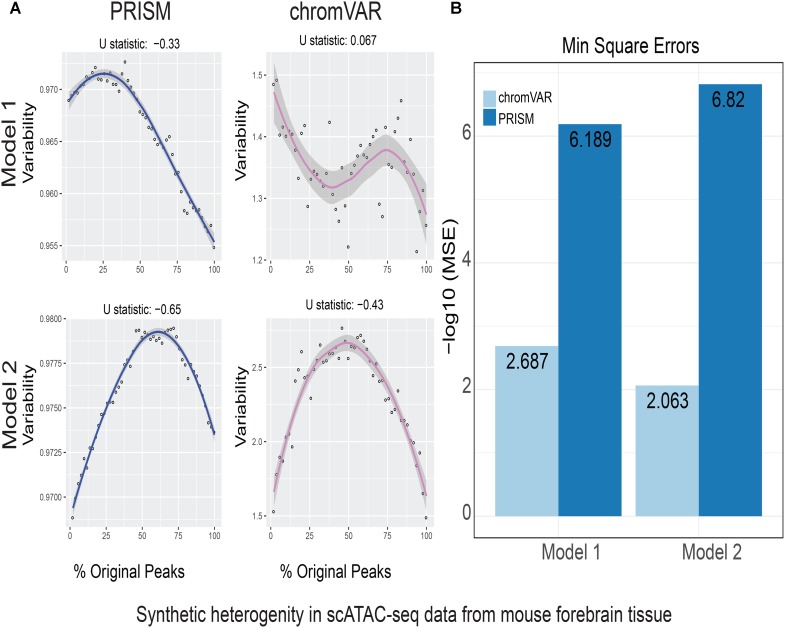
Simulations of cell-to-cell heterogeneity in mouse forebrain tissue. PRISM outperforms chromVAR for data generated under two models. **(A)** In model 1 subtype A, chromVAR does not conform to an inverse-U shape while PRISM does. In model 2 subtype A, chromVAR deviates from the curve of best fit more than PRISM. In order to see how well a simulation fit an inverse-U shape (concave curve), a test of concavity (U statistic) was designed. The difference between variability of successive proportions of cells expressing original peaks was calculated. Then the Spearman correlation of this ordering with the decreasing number sequence 49 through 1 was calculated. This can be seen as checking to see if the derivative (slope) is continuously decreasing. Values close to 1 are ideal. **(B)** PRISM’s measurements were also significantly less noisy (stochastic) compared to chromVAR. To measure noise, we calculated the mean squared error (MSE), or average squared distance of each point from the LOESS curve. PRISM showed orders of magnitude smaller MSE values. The MSE is plotted on -log10 scale.

We synthesized heterogeneity in chromatin accessibility across single cells following two models. In model 1, we assumed comparable levels of total accessibility between individual cells at a set of genomic regions. To recapitulate conditions favorable to chromVAR’s assumptions, we developed model 2 and incorporated differences in total accessibility among cells. To model the genomic regions of interest, we randomly selected 500 (or 1000) peaks from ∼50,000 open chromatin regions. Relying on the central limit theorem, the randomly selected original and GC-matched peaks in model 1 comprise comparable levels of average accessibility across individual cells. Simulated heterogeneity is then created by titrating the number of cells with the original or GC-matched peaks. This approach leads to a mixture of cells containing varying percentages of original versus GC-matched peaks (**Figure 2A**). The rationale to mix peaks rather than cells of different types is to prevent confounding factors such as differences in cell lysis affecting our assessment of cell-to-cell variability. We expect that when cells contain only original peaks (or GC-matched peaks), the variability should be at a minimum. In contrast, when half of cells contain original peaks, the variability should be at a maximum. Based on how mixing of cells is controlled, we expect an inverse-U or concave shape for our measure of variability, peaking at around 50–50 mixed peaks.

We further synthesize heterogeneous data using model 2 relying on the same approach except that GC-matched peaks are drawn from peaks with greater than 75th percentile in mean accessibility compared to all other peaks. In other words, model 2 assumes the presence of a significant difference in total accessibility between cells underscoring a larger degree of cellular heterogeneity. We further augmented each model to have subtypes A and B such that subtype A utilized cells with highest accessibility in contrast with subtype B that relied on lowest accessible cells. While subtype A is the most robust measurement and reflects an ideal sequencing coverage, subtype B tests the method’s sensitivity to technical noise. Together, model 1 is built such that heterogeneity is not caused by differences in total accessibility between cells, simulating cases where an ensemble of cells contains comparable total accessibility levels across the genome but accessibility can occur at completely different regulatory elements (**Supplementary Figure S1**). On the other hand, model 2 aims to simulate cases where a major difference exists in the total accessibility of cells at genomic regions of interest (**Supplementary Figure S1**).

In order to see how well a simulation result fits an inverse-U shape (concave curve), a test of concavity was designed. The metric, referred to as U statistic, measures the difference between variability of successive proportions of cells containing the original peaks. Then the Spearman correlation of this ordering with the decreasing numbers 49 through 1 was calculated. This procedure checks if the derivative (slope) is continuously decreasing. Using this definition, test of concavity values (U statistic) close to 1 are ideal. We also measured each algorithm’s mean squared error (MSE) from its local polynomial regression (LOESS) curve. This assessed the degree to which an algorithm was susceptible to random fluctuations or noise. We further compared the values of variability across the simulated heterogeneous data as measured by chromVAR (Schep et al., 2017).

### Publicly Available Single-Cell Chromatin Accessibility Data

The raw files for scATAC-seq data were generated in mouse forebrain tissue at E11.5 (GSM2668117) (Preissl et al., 2018), mouse double-positive thymocytes (GSE99159) (Johnson et al., 2018), and human AML cells (GSE96769) (Corces et al., 2016). Single-cell accessibility data and ChIP-seq data for 139 transcription factors in K562 cell line were downloaded from ENCODE.

## Results

### The PRISM Algorithm

We introduce PRISM for estimating cell-to-cell variation at the level of chromatin accessibility. Unlike chromVAR, PRISM infers cell-to-cell variability by capturing differences in accessibility of every genomic region of interest across individual cells. It is possible that a comparable total number of accessible sites occur in a cell, yet accessibility happens at non-overlapping regulatory elements (**Supplementary Figure S1**). Our algorithm takes binarized read counts of individual cells and calculates variation of open chromatin across single cells at DNA sequences unified by an annotation such as transcription factor binding events or enrichment of motifs (i.e., a set of peaks). PRISM then represents each cell in a high-dimensional space as a vector (**Figure 1**). Each coordinate in the vector corresponds to accessibility of a regulatory element. To measure how different any two cells are at a given group of genomic regions, PRISM calculates the pair-wise angular cosine distance or the angle between two vectors (**Figure 1**). The pair-wise differences between cells are then used to measure how different every cell is from the group “average”: Each cell is plotted as a point in principal coordinate space such that the Euclidean distance between two points (cells) is equal to the original angular cosine distance between two vectors. PRISM then finds the centroid of this unique point configuration. Every point’s distance from the centroid is calculated, and then these distances are averaged. This can be seen as each cell’s distance from the group norm for chromatin accessibility and constitutes our measure of cell-to-cell variation prior to technical bias correction (**Figure 1A**). Our proposed method scales linearly with heterogeneity, in contrast to average angular cosine distance.

To account for technical biases, a user-defined number of “background” sets of peaks are identified for every set of genomic regions. The background peak sets are matched for peak number, overall mean accessibility, and peak-for-peak GC content to the original peak set. Using the procedure outlined above, accessibility variation for each background peak set is calculated. The variations of the background sets are then averaged. To obtain the bias-corrected variation, the variation of the original peak set is divided by the average variation of the background peak set with matching mean accessibility and GC content. After correcting for technical biases, a negative control is developed: a user-defined number of sets of peaks are randomly selected, each with equal peak number to the original peak set. The bias-corrected variation of each negative control peak set is calculated. Then the bias-corrected variation of the original peak set is divided by the average of the user-defined number of negative control peak sets. This measures cell-to-cell variation in chromatin accessibility in units of background noise. A calculated variation equal to 1 implies that a chromatin feature is associated with equal variation to background noise. Together, unlike the previously proposed method chromVAR (Schep et al., 2017), which relies on differences in the aggregate of accessibility across a set of peaks between cells, PRISM takes differences in accessibility at each genomic region between single cells into account.

### Evaluating PRISM by Simulating Heterogeneity in Single Cell Chromatin Accessibility

To benchmark the performance of PRISM and chromVAR, we simulated heterogeneity in single-cell chromatin accessibility data. We exploited three independent publicly available datasets generated by two leading experimental protocols, that is, the automated microfluidic platform (Buenrostro et al., 2015; Corces et al., 2016; Johnson et al., 2018) and the combinatorial indexing assay (Cusanovich et al., 2015, 2018; Preissl et al., 2018). The data sets used for our study were generated in acute myeloid leukemia (AML) cells in humans (Cusanovich et al., 2015), in addition to double-positive T cells in the thymus (Johnson et al., 2018) and forebrain tissue (Preissl et al., 2018) in mice.

**FIGURE 3:**
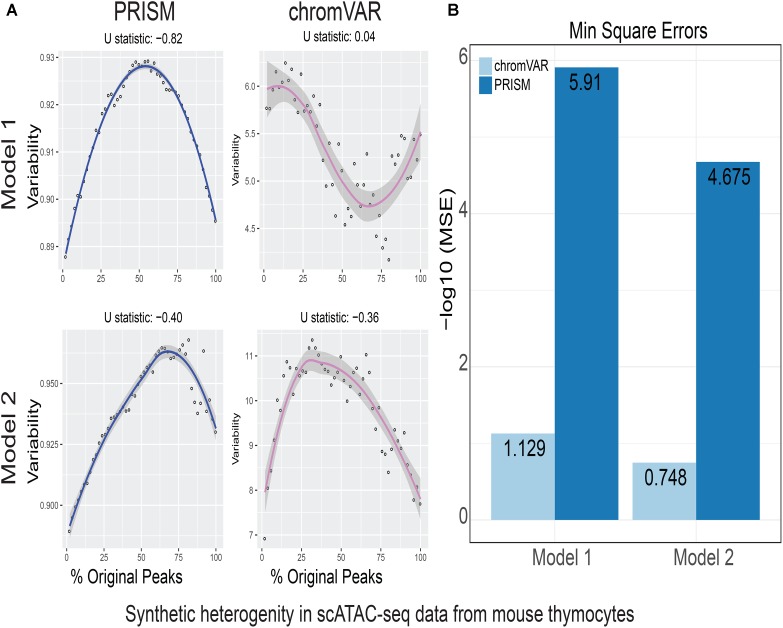
Simulations of cell-to-cell heterogeneity in mouse double-positive T cells. PRISM outperforms chromVAR for data generated under two models when heterogeneity was generated for mouse double positive T cells (Johnson et al., 2018). **(A)** In model 1 subtype A, chromVAR does not conform to an inverse-U shape while PRISM does. In model 2 subtype A, chromVAR deviates from the curve of best fit more than PRISM. In order to see how well a simulation fit an inverse-U shape (concave curve), a test of concavity (U statistic) was designed. The difference between variability of successive proportions of cells expressing original peaks was calculated. Then the Spearman correlation of this ordering with the decreasing number sequence 49 through 1 was calculated. This can be seen as checking to see if the derivative (slope) is continuously decreasing. Values close to 1 are ideal. **(B)** PRISM’s measurements were also significantly less noisy (stochastic) compared to chromVAR. To measure noise, we calculated the mean squared error (MSE), or average squared distance of each point from the LOESS curve. PRISM showed orders of magnitude smaller MSE values. The MSE is plotted on -log10 scale.

**FIGURE 4:**
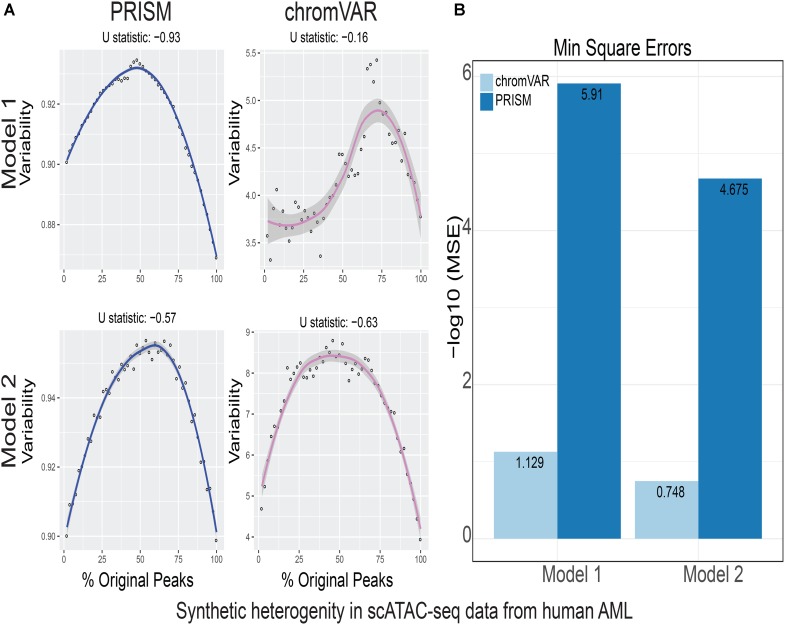
Simulations of cell-to-cell heterogeneity in human AML cells. PRISM outperforms chromVAR for data generated under two models when heterogeneity was generated for human AML cells (Corces et al., 2016). **(A)** In model 1 subtype A, chromVAR does not conform to an inverse-U shape while PRISM does. In model 2 subtype A, chromVAR deviates from the curve of best fit more than PRISM. In order to see how well a simulation fit an inverse-U shape (concave curve), a test of concavity (U statistic) was designed. The difference between variability of successive proportions of cells expressing original peaks was calculated. Then the Spearman correlation of this ordering with the decreasing number sequence 49 through 1 was calculated. This can be seen as checking to see if the derivative (slope) is continuously decreasing. Values close to 1 are ideal. **(B)** PRISM’s measurements were also significantly less noisy (stochastic) compared to chromVAR. To measure noise, we calculated the mean squared error (MSE), or average squared distance of each point from the LOESS curve. PRISM showed orders of magnitude smaller MSE values. The MSE is plotted on -log10 scale.

We calculated variability of chromatin accessibility at the single-cell level across the simulated sets of peaks using PRISM and chromVAR. In data generated by combinatorial indexing technique in thousands of cells in mouse forebrain tissue (Preissl et al., 2018), we found an inverse-U shape in variability for the two models using PRISM with 30 iterations for background peak selection when scATAC-seq data from the mouse forebrain tissue were used (Preissl et al., 2018) (**Figure 2A**). However, chromVAR faltered under model 1 in the case that total accessibility was comparable across cells with a very low test of concavity *U* = 0.067. In model 2, PRISM also conformed better to an inverse-U curve than chromVAR (0.65 vs. 0.43). Notably, PRISM was significantly less noisy, with a mean-square-error (MSE) between the fitted curve several orders of magnitude lower than chromVAR (6 × 10^-7^ vs. 0.5) (**Figure 2B**). We observed similar results when 40 or 50 iterations for background peaks were used for normalization (**Supplementary Figure S2**). PRISM further outperformed chromVAR in cells with the lowest accessibility levels recapitulating noisier sequencing conditions (**Supplementary Figure S3**). These differences were reproduced under both models when the simulated heterogeneity was evaluated for scATAC-seq data generated in hundreds of double-positive T cells from mouse thymus or AML cells in humans using the microfluidic technology (**Figures 3**, **4**). Together, PRISM outperforms chromVAR in assessing variability of chromatin accessibility at the single-cell level across multiple scATAC-seq datasets.

### PRISM and ChromVAR Differ in Predicting Cell-to-Cell Variability in Biological Data

**FIGURE 5:**
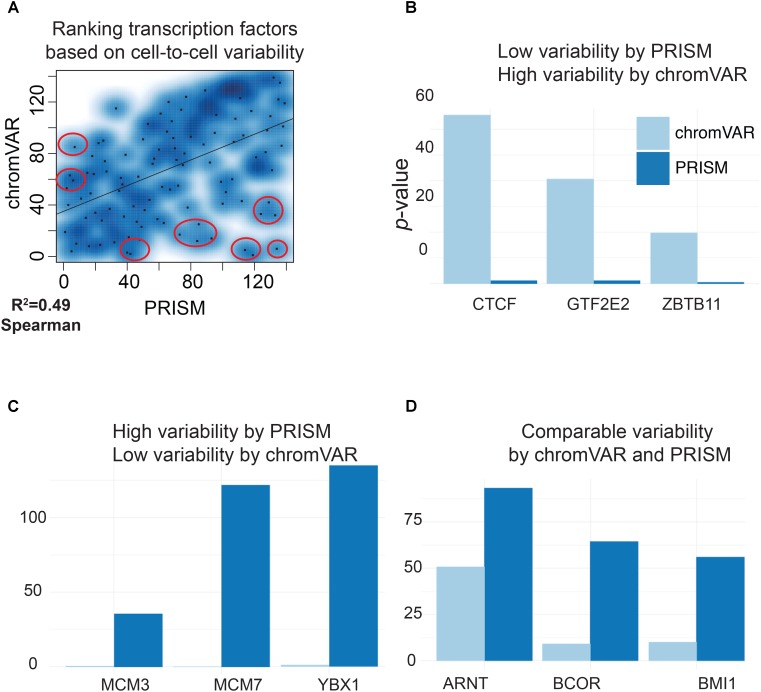
PRISM reveals previously masked chromatin accessibility variation on K562 cells. **(A)** Transcription factor binding data were extracted for K562 cell line using the ENCODE data. chromVAR and PRISM are consistent in inferring cell-to-cell variability in 122 transcription factors while predictions are different for 17 transcription factors (*R* = 0.49). **(B)** We found six transcription factors with high rank of variability by chromVAR but low rank by PRISM. Three of these TFs including CTCF are shown. **(C)** Three transcription factors (MCM3, MCM7, and YBX1) were in the upper 25th percentile in variability for PRISM, but found neutral by chromVAR. **(D)** PRISM and chromVAR were consistent on the majority (122/139) of transcription factors examples include ARNT, BCOR, and BMI1.

We next compared the predictions of PRISM and chromVAR on the effect of 139 transcription factors using real transcription factor binding data. Assessing cell-to-cell variability using the two methods revealed different predictions for 17 transcription factors in K562 cell line (**Figure 3A**). Among transcription factors that were reported differently between two methods, chromVAR but not PRISM inferred that CTCF binding events in K562 cell line could increase cell-to-cell variability at the chromatin accessibility level (**Figure 3B**). However, numerous studies mapping the genome-wide binding events of CTCF at the population level across a wide variety of tissues have shown the cell-type-invariant binding of this protein acting as an insulator, supporting PRISM’s prediction (Schmidt et al., 2012). On the other hand, several transcription factors including MCM family proteins were associated with high cell-to-cell variability by PRISM in contrast with chromVAR. Of note, the two methods were consistent in assessing variability at binding events of majority of transcription factors (**Figure 5**). Together, our simulation results titrating heterogeneity of chromatin accessibility at the single-cell level together with the application of biological data suggest that PRISM can infer cell-to-cell variability on the chromatin at the single-cell level and outperforms the existing method chromVAR.

## Discussion

Since the discovery of the cell by Robert Hooke in 1665, biologists and pathologists have been fascinated by the diversity of cell types in our body. With the advent of molecular cell biology, methods such as fluorescent protein reporters and single-molecule detection of RNA or DNA have been developed for measuring properties and functions of single cells at increasing resolution (Kelsey et al., 2017). The utilization of these techniques in multiple organisms revealed that gene expression is a fundamentally stochastic process, with randomness in transcription and translation leading to cell-to-cell variations in mRNA and protein levels (Raj and van Oudenaarden, 2008; Symmons and Raj, 2016). But how can the reported stochastic gene expression be reconciled with the determinism of cellular development? We postulate that development is controlled by lineage-determining transcription factors, which as their name implies confer less stochasticity in individual cells. We recently generated scATAC-seq in double-positive T cells and measured cell-to-cell variability using normalized cosine distance. We found that the lineage-determining transcription factor TCF-1 is associated with low level of variability across individual cells following T cell trajectory (Johnson et al., 2018). In this work, we presented our proposed computational approach in detail along with an R package and comprehensively compared this technique with the existing method chromVAR. chromVAR aggregates chromatin accessibility across peaks that share a common feature and assess the variability of the aggregate of accessibility between individual cells. While the aggregation of signal may address the variability of some single-cell data sets with certain statistical properties, this approach inherently masks heterogeneity within genomic regions across individual cells. To address this limitation, we developed PRISM a linear algebra-based method that takes into account the differences between every cell pair at individual genomic regions while correcting for GC-bias and average accessibility.

To evaluate the performance of PRISM and compare it with chromVAR in assessing variability of chromatin accessibility across single cells, we devised a computational experiment and generated heterogeneity in chromatin accessibility by shuffling scATACseq data. Our framework generated simulated scATAC data from the real measurements with various degrees of heterogeneity, which were further used to evaluate the performance of PRISM. While variability evaluated by PRISM increased as heterogeneity increased, chromVAR failed to perform in cases where heterogeneity existed between peaks and across cells as a result of aggregating signal across all peaks (in particular model 1). We further showed that PRISM but not chromVAR can predict CTCF binding events to associate with low level of variability across individual cells.

We have shown that our method, named PRISM, is able to overcome the obstacles in analyzing single-cell ATAC-seq, caused by the inherent nature of such assays, and provide a robust framework that assesses the effects of transcription factor binding on the chromatin accessibility, at the single-cell level. When compared to the state-of-the-art method, named chromVAR, we have shown that PRISM facilitated the discovery of lineage-determining transcription factors with the ability to preserve low variability of chromatin accessibility at the single-cell level.

## Data Availability

The datasets GSE99159 for this study can be found in the NCBI GEO. PRISM is an open source framework, freely accessible through Github (https://github.com/VahediLab/PRISM).

## Author Contributions

All authors contributed extensively to the work presented in this paper. GV conceived the project, administered the analyses, and wrote the manuscript. SC developed and implemented the method. GG and JJ provided technical support and conceptual advice.

## Conflict of Interest Statement

The authors declare that the research was conducted in the absence of any commercial or financial relationships that could be construed as a potential conflict of interest.

## Supplementary Material

FIGURE S1 Chromatin accessibility variation can exist even if total accessibility is the same between cells. Measurements of an ensemble of cells can show similar total level of accessibility within cells (i.e., comparable number of accessible sites in a cell) yet accessibility can occur at non-overlapping regulatory elements. In this hypothetical case, red represents an accessible sequence in a given cell, and blue represents an inaccessible sequence. Each cell has three accessible sequences (red) total, or a total accessibility of 3. Thus, the total accessibility is the same between cells. But each cell is accessible at completely different DNA sequences, which may have different functions. Existing algorithms, such as chromVAR and ((s))Buenrostro et al. (2015) workflows, are built on the standard deviation of total accessibility, hence they cannot measure variation in these cases.

FIGURE S2 PRISM outperforms chromVAR for multiple values of peak number for background peaks. PRIMS outperforms chromVAR when 40 or 50 background peaks are selected in calculating variability in mouse forebrain tissue, mouse double-positive T cells and human AML cells.

FIGURE S3 PRISM outperforms chromVAR under subtype B when cells with low chromatin accessibility are selected. PRISM outperforms chromVAR under subtype B when cells with low chromatin accessibility are selected in mouse double-positive T cells and human AML cells.
