# Supplementary figures and images for: A Cosine Similarity-Based Method to Infer Variability of Chromatin Accessibility at the Single-Cell Level
Source: Front Genet. 2018 Aug 15;9:319. doi: 10.3389/fgene.2018.00319 (PMC6103536; doi:10.3389/fgene.2018.00319)

# Figure S1

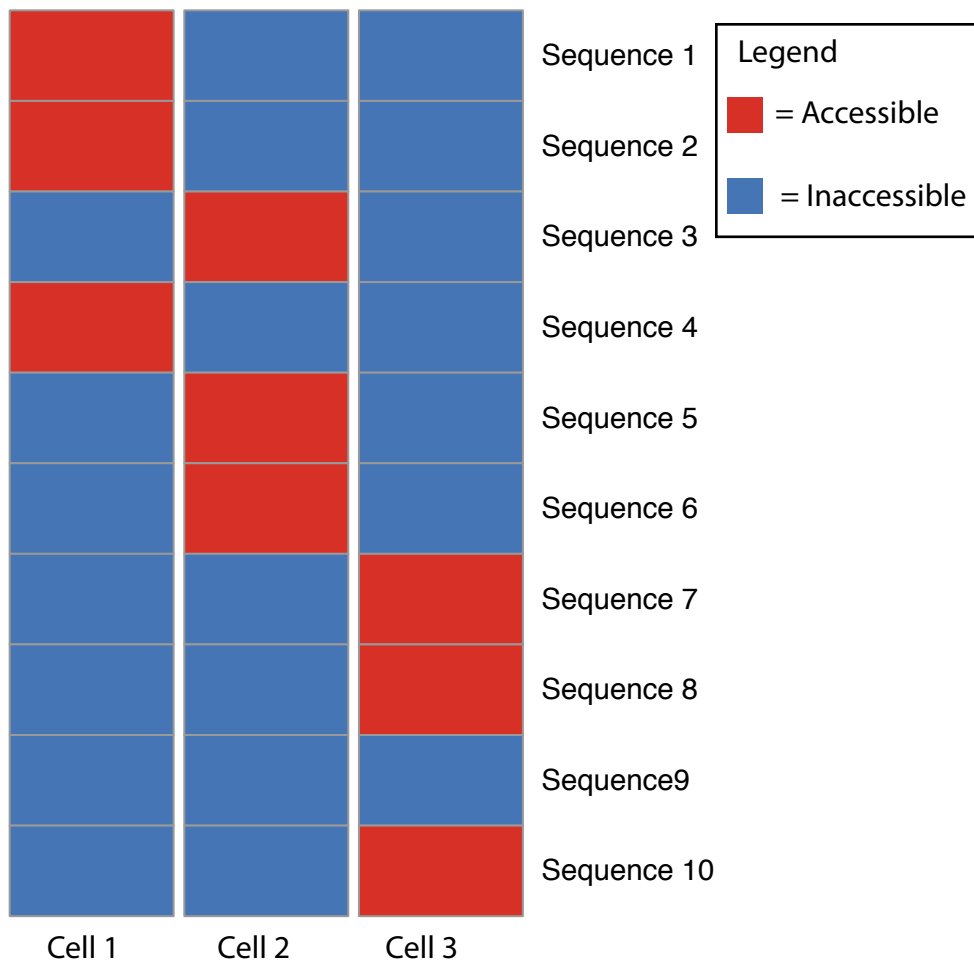

Supplement: FIGURE S1 — Chromatin accessibility variation can exist even if total accessibility is the same between cells. Measurements of an ensemble of cells can show similar total level of accessibility within cells (i.e., comparable number of accessible sites in a cell) yet accessibility can occur at non-overlapping regulatory elements. In this hypothetical case, red represents an accessible sequence in a given cell, and blue represents an inaccessible sequence. Each cell has three accessible sequences (red) total, or a total accessibility of 3. Thus, the total accessibility is the same between cells. But each cell is accessible at completely different DNA sequences, which may have different functions. Existing algorithms, such as chromVAR and ((s))Buenrostro et al. (2015) workflows, are built on the standard deviation of total accessibility, hence they cannot measure variation in these cases. [file Image_1.pdf]

Fig S2

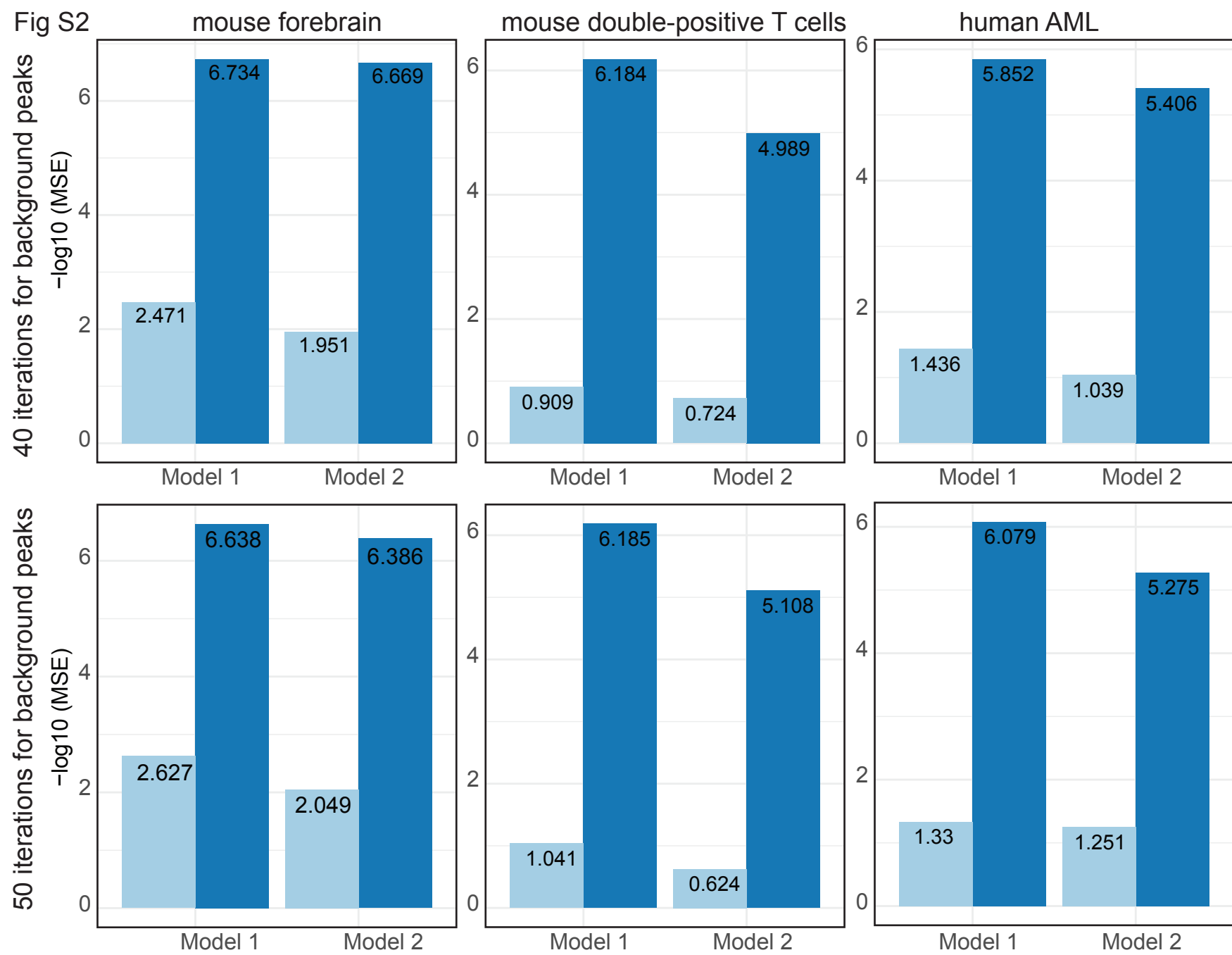

Supplement: FIGURE S2 — PRISM outperforms chromVAR for multiple values of peak number for background peaks. PRIMS outperforms chromVAR when 40 or 50 background peaks are selected in calculating variability in mouse forebrain tissue, mouse double-positive T cells and human AML cells. [file Image_2.pdf]
