# Supplementary material for: A Cosine Similarity-Based Method to Infer Variability of Chromatin Accessibility at the Single-Cell Level
Source: Front Genet. 2018 Aug 15;9:319. doi: 10.3389/fgene.2018.00319 (PMC6103536; doi:10.3389/fgene.2018.00319)
Supplement: FIGURE S3 — PRISM outperforms chromVAR under subtype B when cells with low chromatin accessibility are selected. PRISM outperforms chromVAR under subtype B when cells with low chromatin accessibility are selected in mouse double-positive T cells and human AML cells. [file Image_3.pdf]

Fig S3

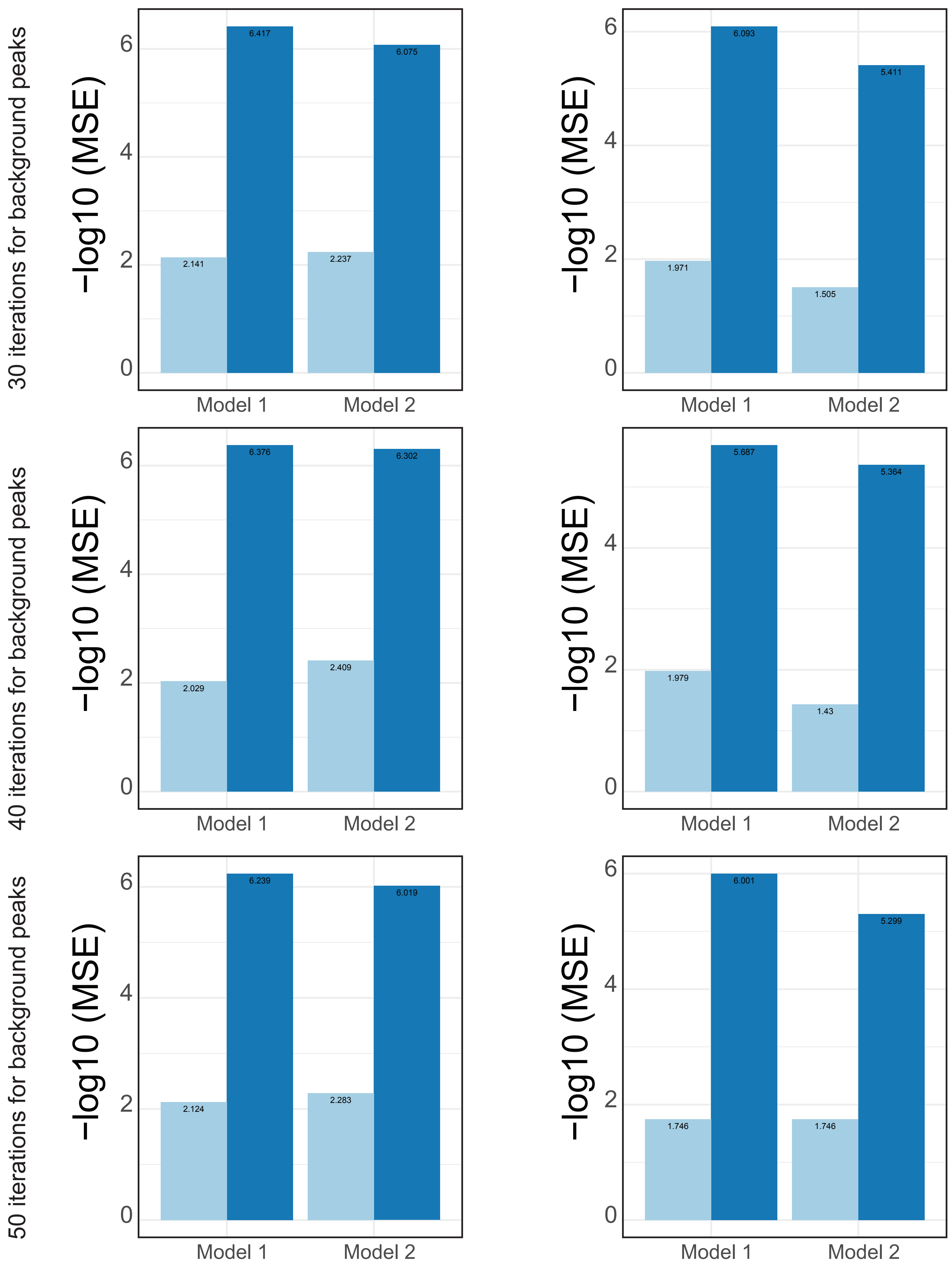
mouse double-positive T cells
human AML
30 iterations for background peaks
40 iterations for background peaks
50 iterations for background peaks
−log10 (MSE)
Model 1
Model 2
2.141
6.417
2.237
6.075
1.971
6.093
1.505
5.411
2.029
6.376
2.409
6.302
1.979
5.687
1.43
5.364
2.124
6.239
2.283
6.019
1.746
6.001
1.746
5.299
